# Heart Failure 2013

**DOI:** 10.1155/2013/342316

**Published:** 2013-12-30

**Authors:** Gregory Giamouzis, Filippos Triposkiadis, Vasiliki V. Georgiopoulou, Dimitrios Farmakis, Dirk Westermann, John Skoularigis

**Affiliations:** ^1^Cardiology Department, Larissa University Hospital, P.O. Box 1425, 41110 Larissa, Greece; ^2^Division of Cardiology, Emory University Hospital, Atlanta, GA 30322, USA; ^3^First Department of Internal Medicine, University of Athens Medical School, Athens, Greece; ^4^Department of General and Interventional Cardiology, University Heart Center Hamburg Eppendorf, Martinistraße 52, 20246 Hamburg, Germany

The heart failure “growing epidemic” is an important public health issue that the health care systems of all developed countries are facing. Over the last decade, the annual number of heart failure hospitalizations has almost doubled with approximately 50% of patients being rehospitalized within 6 months of discharge [1]. The complex array of physiologic, psychological, social, and health care delivery issues makes it a challenging chronic disease to manage. To implement cost-effective strategies to curb the epidemic, better understanding of the underlying pathophysiological mechanisms as well as novel diagnostic and therapeutic approaches is needed.

In this special issue we have invited a few papers that address such issues and explain why, despite the emergence of novel therapeutic approaches that promise life prolongation and hospital length reduction, this patient population will still be needing rehospitalization and will often have a poor prognosis. This special issue is the extension of an effort that was initiated in 2011 with the first heart failure-focused issue, [2] coupled with a second focused issue a year later [3].

We have divided this issue into 4 categories: the pathophysiology section, the pharmacological treatment section, the nonpharmacological treatment section, and the comorbidity section.

According to the ejection fraction, patients with heart failure have traditionally been divided into two different groups: heart failure with preserved and heart failure with reduced ejection fraction. In recent years, accumulating studies showed that increased mortality and morbidity rates of these two groups are nearly equal. Although heart failure with preserved ejection fraction (formerly called “diastolic heart failure”) is an increasingly frequent condition of heart failure worldwide, its pathophysiology has not been sufficiently elucidated. This is thought to be the most significant reason for a lack of established treatment methods in this patient population. In the *pathophysiology section*, K. Komamura and colleagues provide a comprehensive review of the similarities and differences between the pathogenesis and pathophysiology of diastolic and systolic heart failure.

Shedding light in a very similar topic, in the *pharmacological section*, J. Li and colleagues focus on the tested as well as the promising therapeutic options that are currently studied in patients with heart failure with preserved ejection fraction and provide a brief discussion on the pathophysiological mechanisms and diagnostic options that are helpful to increase our understanding of these novel therapeutic strategies. In an original study, A. Orea-Tejeda et al. evaluate the effect of ivabradine on endothelial function in diastolic and right heart failure patients. Ivabradine is an *I*(*f*) ion current inhibitor that has proved to reduce mortality in patients with systolic heart failure by slowing heart rate without decreasing myocardial contractility but has not been tested in patients with preserved ejection fraction. Diastolic and right heart failure patients underwent photoplethysmography, a simple, low-cost optical technique that can evaluate vascular function, before and after induced ischemia and before and 6 months after treatment with ivabradine (mean 12.5 mg a day). Ivabradine administration significantly improved endothelial function (shear stress) in this patient population.

The accurate impact of exercise on coronary artery disease patients with left ventricular dysfunction is still debatable. In the *non-pharmacological treatment section*, M. Khosravi and colleagues study the effects of cardiac rehabilitation on echocardiography parameters in coronary artery disease patients with left ventricular dysfunction. Following an eight-week exercise-based rehabilitation program, all subjects significantly increased their left ventricular ejection fraction and peak exercise capacity without experiencing any serious cardiac complication.

A shared understanding of medical conditions between patients and their health care providers has been shown to improve self-care and outcomes [4]. In the *comorbidity section*, we demonstrate how certain comorbid conditions may affect patients' decision-making capacity and interfere with their ability to comply with treatment requirements, recognize and self-manage disease worsening symptoms [5]. Sleep disordered breathing, encompassing both obstructive and central sleep apnea, has been associated with increased cardiovascular morbidity and mortality. It occurs in almost half of all heart failure patients and is linked to hypertension, arrhythmia, impaired glucose tolerance, cerebrovascular disease, and ischemic heart disease. Despite the high prevalence and significant morbidity associated with this comorbid condition, our awareness and understanding of sleep disordered breathing remain incomplete [6]. E. Stopford and colleagues outline the available evidence linking sleep disordered breathing and cardiovascular disease and discuss the potential consequences and management in the heart failure population in particular.

We hope that the readers of the journal will find the topics as interesting and important as we did.

*Gregory  Giamouzis* *Filippos  Triposkiadis* *Vasiliki  V.*  *Georgiopoulou* *Dimitrios  Farmakis* *Dirk  Westermann* *John  Skoularigis*
